# Introduction: The Politics of Diversity in Music Education

**DOI:** 10.1007/978-3-030-65617-1_1

**Published:** 2021-03-20

**Authors:** Alexis Anja Kallio, Kathryn Marsh, Heidi Westerlund, Sidsel Karlsen, Eva Sæther

**Affiliations:** 11 Queensland Conservatorium, Griffith University, Brisbane, QLD Australia; 12 Sibelius Academy, University of the Arts Helsinki, Helsinki, Finland; 13 Music Education and Music Therapy Department, Norwegian Academy of Music, Oslo, Norway; 14 Sydney Conservatorium of Music, University of Sydney, Sydney, NSW Australia; 15 Malmö Academy of Music, Lund University, Lund, Sweden; 16 Queensland Conservatorium, Griffith University, Brisbane, QLD Australia; 17 Sydney Conservatorium of Music, University of Sydney, Sydney, Australia; 18 Sibelius Academy, University of the Arts Helsinki, Helsinki, Finland; 19 Music Education and Music Therapy Department, Norwegian Academy of Music, Oslo, Norway; 20 Malmö Academy of Music, Lund University, Lund, Sweden

## Abstract

*The Politics of Diversity in Music Education* attends to the political structures and processes that frame and produce understandings of diversity in and through music education. Recent surges in nationalist, fundamentalist, protectionist, and separatist tendencies highlight the imperative for music education to extend beyond nominal policy agendas to critically consider the ways in which understandings about society are upheld or unsettled and the ways in which knowledge about diversity is produced. This chapter provides an overview of the scholarly foundations that this book builds upon before introducing the four sections of the book and contributing chapters. The first section of the book focuses on the politics of inquiry in music education research. The second section attends to the paradoxes and challenges that arise as music teachers negotiate cultural identity and tradition within the political frames and ideals of the nation state. The third section considers diversities that are often overlooked or silenced, and the final section turns to matters of leadership in higher music education as an inherently political and ethical undertaking. Together, chapters work towards a more critical, complex, and nuanced understanding of the ways in which the politics of diversity shape our ideals of what music education is, and what it is for.

## Introduction

*The Politics of Diversity in Music Education* attends to the political structures and processes that frame and produce understandings of diversity in and through music education practice, policy, and research. With the contemporary, globalized world characterized by intense mobility, mass migration, and fast-paced advances in technology and communication, music education is in a unique position to (re)consider the “modes of cultural confluence… and the ways in which individuals in complex settings relate to each other from different vantage points” (Vertovec 2010, p. 67). Recent surges in nationalist, fundamentalist, protectionist, and separatist tendencies pose a heightened imperative for music education to engage with diversity, particularly with regard to the ways that education contexts such as schools or universities uphold or unsettle understandings about society and the ways in which knowledge about diversity is produced. Accordingly, critical analyses of *diversity* in music education scholarship have not only drawn attention towards who is *included* (or excluded) as part of teaching and learning but has also framed diversity as a normative expression: a value to which all institutions, teachers, and students ought to be committed.

Although many music education policies outline an explicit agenda for diversity, little attention has been paid to the complex situations that arise when negotiating diversity in practice. *The Politics of Diversity in Music Education* aims to remedy this knowledge gap by critically attending to the ways in which difference is promoted, represented, negotiated, navigated, contained, or challenged in various music education practice, policy, and research contexts. Diversity, here, is not a label applied to certain individuals or musical sounds and repertoires per se but is rather understood as socially organized difference, produced, and manifested in various ways as part of complex relations and interactions between people and social groups. Thus, the aim of this book is not to fortify the categorization of people and their musics but to focus on the power relations that are “inherent in the constitutive conditions of differences and constantly (re-)produced, shifted and thereby potentially transformed by every act of differentiation” (Dobusch 2017, p. 1648). The *politics* at hand are thus not those concerning politicians acting *for* the people or those relating to the political functions and roles of musics as part of public protest, for example (see Hesmondhalgh 2013). Rather, the politics of diversity here refers to the everyday processes by which we all exercise agency, negotiate power and identity, and assign meaning to difference.

This book builds upon a legacy of scholarship and practice that has positioned education as an important arena for social change, cultural change, and ethical practice. One of the most well-established and enduring developments towards social transformation through education is *multicultural education.* As James A. Banks wrote already in 1993, “[m]ulticultural education… is a movement designed to empower all students to become knowledgeable, caring, and active citizens in a deeply troubled and ethnically polarized… world” (p. 23). In music education, the late 1980s and early 1990s witnessed an important shift in terms of what repertoires were taught in school and university classrooms and what for. Music education was seen as an arena wherein *all* learners could be engaged as a community, by both bringing people together through musical practice and heightening their intercultural sensitivities. For example, Keith Swanwick (1988) suggested an intercultural approach that holds the potential to “reduce the power of [cultural] stereotypes” (p. 4) through cultivating an awareness of the “universality of musical practice” (p. 8) and the unique sonic beauty of different musical traditions through the creation of “new values and transcending both self and social culture” (p. 6). Comparing music to language, Swanwick (1994) argued that “it is nonsense to say that we cannot understand music without understanding the culture from which it came. The music *is* the culture” (p. 222). In this sense, teachers were directed to approach music as a universal phenomenon that in itself holds the potential to exist distinct from sociocultural context or social ties and rise above the power relations relating to the politics of diversity that arise in any given education context. Scholars working at the intersection of music education and ethnomusicology challenged this understanding, as Anthony Palmer (1992) argued, “artistic expression weakens when it becomes generalized. One thing that we must learn about art is its undeniable and crucial need for specificity” (p. 35). Accordingly, while music *was* seen as a “pan-human” experience, it was also positioned as a “culture-specific” practice (Campbell 2017, p. 16; Volk 1998) warranting particular considerations when transferred from original settings to education contexts. Some scholars advised teachers to work to preserve the authenticity of musical expressions (Elliott 1995), while others emphasized the inevitability of “recontextualization” (e.g. Schippers 2010; Määttänen and Westerlund 2001).

As the attention of ethnomusicologists has focused more on issues such as “identity, representation, nationalism, gender, diaspora, globalisation, human and cultural rights, and education” (Pettan 2009, p. 56), the political nature of music and musical participation has been understood in increasing complexity with regard to questions of culture, social change, and ethics. For instance, discourses in ethnomusicology and music education have shifted from “realist assumptions of authenticity” to authenticity as a “socially constructed phenomenon” (Vannini and Williams 2009, p. 2; Kallio et al. 2014) and from easily recognizable borders between insiders and outsiders to a more blurred and dynamic conception of the *borders* and *boundaries* (Campbell 2018) that define the mainstream and the margins. As Patricia Shehan Campbell (2018) has argued, “[m]usic is a powerful means of defining heritage, developing intercultural understandings, and breaking down barriers between various ethnic, racial, cultural and language groups,” also holding “potential to impact… the knowledge construction process, prejudice reduction, an equity pedagogy, and an empowering school culture” (p. xvi). In higher music education, intercultural projects have consciously challenged once taken-for-granted professional boundaries and understandings of what it means to study music at the tertiary level, with collaborations established between institutions, with students, as well as together with underserved communities (Marsh et al. 2020). Further, practice-based research at the intersection of music education and ethnomusicology has underlined the importance of societal networks and expanded notions of professionalism (Sæther 2020), highlighted the inherently unpredictable nature of intercultural collaboration and the need for flexibility (Westerlund et al. 2015). Such partnerships have called into the question the underlying values and fundamental principles upon which music teacher education is based, raising questions as to who higher music education serves and to what ends (Kallio and Westerlund 2020).

Related to such critical perspectives, music education scholars have problematized the conditions that give rise to music’s potential to promote intercultural understanding and equity in light of contemporary individual and social experience. For example, Karlsen (2017) notes that “access to a multicultural education experience seems to depend on the existence of ‘roots,’ understood as individuals acknowledging that they in fact do belong to specific cultural traditions that can either be moved beyond, strengthened, or understood as processes” (p. 216). Similarly, Hess (2015) explains that multicultural music education can itself serve as a mechanism of inequality, positioning the majority culture as the neutral core of the curriculum while arranging “so-called ‘other’ musics… around its periphery” (p. 338). Indeed, Westerlund et al. (2020; also Karlsen and Westerlund 2015 and Westerlund, Partti and Karlsen 2017) argue that the provision of appropriate music education repertoires and approaches according to categories derived from peoples’ geographical and ethnic backgrounds is overly simplistic, increasingly irrelevant, and possibly fallacious, resulting in the essentialization of identities, the reification of dominant hegemonies, and the reinforcement of inequity. Contemporary understandings of culture as multiple and fluid (Bauman 2010), combined with the normative and critical ideals of any education not just to describe and reproduce culture but also to enact positive sociocultural and political change, is at the heart of the increasing need to rethink the politics of diversity in music education. An example of this can be seen in the life story of a Newar musician from the Kathmandu Valley (Westerlund and Partti 2018) who is at once a culture-bearer concerned with the protection and sustainability of his musical heritage but also a committed cosmopolitan activist working towards radical social change and transformation. Hence, as Westerlund and Karlsen (2017) explain, we must do more than diversify our repertoires or pedagogies in order to engage ethically and meaningfully with our students and consider “the ethics, politics, and ideologies of diversity that condition our understanding of diversity itself” (p. 100). Professionalism in music education can thus be seen as a moral commitment to “understand our relationships (in music education) as under construction” and a turn to *responsibility*, while constantly “reflecting what responsibility means” (Westerlund 2019, p. 513). In moving beyond the good intentions and visions of diversity in music education that foreground togetherness and harmony, in this book we recognize that learning and practicing “the art of living with difference” (Bauman 2010, p. 151) is a complex process that is always in the making. Furthermore, as a process inherently bound with societal transformation and institutional change, we position music education as a social and political arena wherein we may productively grapple with uncertainty, conflict, and change in working towards mutual respect without necessarily reaching mutual agreement.

*The Politics of Diversity in Music Education* includes and extends recent work conducted within the Academy of Finland funded research project *Global Visions Through Mobilizing Networks: Co-developing Intercultural Music Teacher Education in Finland, Israel and Nepal*, by broadening the critical and collaborative deliberations of diversity in music education to a broad array of disciplinary perspectives and sociocultural contexts. While a number of the editors were associated with this project, the idea of such a collection was initiated at the *13th Cultural Diversity in Music Education Conference* in Kathmandu in 2017, where presentations and ensuing discussions challenged contemporary understandings of diversity in music education scholarship. The “mobilizing network” (Ball and Tyson 2011, p. 412; Darling-Hammond and Lieberman 2012) of the project was thus enacted, bringing together an editorial team from different geographical regions and areas of scholarly expertise. Contributions to the volume were sourced through an open call for chapters, seeking critical perspectives on the politics of diversity from a variety of scholarly and geographical standpoints, thus contributing to “networked expertise” (Hakkarainen 2013). Chapters attend to the politics of diversity as conceptualized and manifest through different theoretical, empirical, and methodological perspectives, in the contexts of higher education, school music lessons, community music programs, curricula, and policy directives, highlighting the international imperative and opportunities for music education to engage with diversity in complex and critical ways. We are mindful that the contributing authors and editorial team, although diverse in many ways, do not adequately represent the diversity of many student populations or communities to whom music educators and music education systems are answerable. Furthermore, a reflexive and critical reading of these chapters and the book as a whole raises important questions that continue to trouble and inspire us, with regard to the Eurocentricity of knowledge production, the political economy of the publishing industry, and the processes by which some of us are able to claim universality and others are relegated to the margins of particularity. This remains as an ongoing practical, theoretical, methodological, ethical and moral task for each of us, and our field more broadly: how can we engage ethically with the politics of diversity when we ourselves are complicit in existing inequities and injustices? Answering and acting upon this question is a shared responsibility for *all* music education scholars and practitioners, and we hope that the critical discussions, new perspectives, reconsiderations, and redirections offered within these pages contribute towards this learning and change.

## Introduction of Chapters

*The Politics of Diversity in Music Education* is structured in four sections. The first of these sections focuses on the politics of inquiry in music education research, inviting the reader to interrogate the processes by which we come to know ourselves and others in music education research and practice. Drawing upon the crisis of representation in anthropology (Marcus and Fischer 1986), postcolonial, and indigenous research perspectives, the authors explore the power dynamics that shape encounters and understandings and the opportunities and limitations of the researcher as instrument. In the initial chapter, **Eva Sæther** searches for “the smell, the groove, [and] the music” in her own ethnographic research through drawing upon the concepts of *radical empiricism* (Jackson 1989) and *sensuous scholarship* (Stoller 1987, 1989). The chapter considers the roles of the body and of music in developing reflexive research methods that take into account different ways of knowing and attend to the complex ethical imperatives of interculturality. This discussion is furthered in **Ailbhe Kenny’s** chapter, which draws on her interactions with asylum seeker children and mothers in Ireland. Understanding the researcher body as political, Kenny offers insights into the multiplicity of positionings for researchers in the field. Troubling the polarity between researcher and researched, she critically explores the process of performing and being recognized as a pregnant researcher in the field, suggesting that researchers ought to reflexively consider the self as “research tool, and thus intimately connected to the methods we deploy” (Cousin 2010, p. 10). The relationality of research practice is further considered in the chapter by **Vilma Timonen**, **Marja-Leena Juntunen**, and **Heidi Westerlund**, as they analyze the politics of reflexivity that emerged through an intercultural collaboration between Finnish and Nepali music teachers. Acknowledging the centrality of reflexivity to deep professional learning, the authors also raise critical questions of power, epistemic imperialism, and coloniality that illustrate the inherently discomfortable and uncertain qualities of reflexive work in cross-national settings. Concluding this section, **Alexis Anja Kallio’s** chapter argues that many enactments of reflexivity in music education serve to reinforce the very inequities they aim to dismantle, “reaffirming the benevolence of the privileged researcher whilst doing little to disrupt the structures that keep such privileges at the center of academic practice.” She invites researchers to consider reflexivity not as a source of superior insight or awareness or a solution to unequal power relations in the research process but as a means to locate opportunities for relational learning and engage critically in the politics of diversity.

The second section of the book attends to the paradoxes and challenges that arise as music teachers negotiate cultural identity and tradition within the changing political frames and ideals of the nation state. Exploring the complexity of teachers’ responses to government mandates in four very different contexts, the chapters shed light on the various ways in which music education might instigate and guide social and political change. The section opens with a chapter by **Michael Webb** and **Clint Bracknell** exploring a paradox that has emerged in Australia’s mainstream music education system, where, despite curriculum mandates, the inclusion of Aboriginal or Torres Strait Islander musics in school music programs has been hampered by teacher apprehension and persistent colonial social structuring in contemporary Australia. Through critically attending to issues of definition, considering the intended audience(s) for Aboriginal and Torres Strait Islander musics, and highlighting pedagogies of partnership and dialogue, the authors argue for approaches that are respectful and mutually enriching and embrace the educative power of indigenous music for all students. In a context with similar policy ideals of cultural inclusion, **Jan Sverre Knudsen** examines the shifting discourses of diversity over three decades of the national Concerts Norway program in schools. He illustrates the ways in which the promotion of diverse music to children can be a component of state policies and state-run development aid, raising important considerations of the ways in which programs are reflective of, and shaped by, musical, multicultural, and political ideologies. **Wai-Chung Ho** highlights the complex negotiations required of teachers in responding to changing state policies and cultural ideals in China. Focusing particularly on the recent push to engage students’ imaginations and cultivate critical thinking skills in the classroom, Ho explores the conflicts that arise between such political directives for creativity and Confucianist educational values that emphasize obedience and order. Through interviews with teachers in Beijing, Ho explores the ways in which music education serves as a locus for the realization of national identity and traditional values, which are not necessarily congruent with each other. This dynamic nature of culture is further explored through the final chapter, by **Danielle Treacy**, **Sapna Thapa**, and **Suyash Neupane** investigating the legitimation of music, music education, and both being and becoming a musician or music teacher in Nepal. In the background of this chapter is a familiar paradox, a society in which music is supported by national policy documents, omnipresent, readily enjoyed, and shared among individuals and social groups but one that also stigmatizes the career of the professional musician. Intensified by a context characterized by extreme and highly complex diversity and a long history of social stratification, the authors illustrate the ways in which musicians and music educators engage in dialogue between aspirations and sedimented traditions as they navigate both the dynamic nature of culture and questions of legitimate knowledge. These negotiations lead to a conceptualization of professionalism in music education beyond musical expertise, to an ethical responsibility encompassing broader questions of how music teacher education might foster a strong and critical sense of non-discrimination and inclusion.

The third section of the book challenges commonly held conceptualizations of diversity in music education as pertaining only to issues of race or ethnicity. Highlighting diversities in music education that are often overlooked or silenced, these chapters raise pertinent questions as to whose difference, and what quality of difference, is considered “diversity-relevant” and by whom (Dobusch 2017). **Minja Koskela**, **Anna Kuoppamäki**, **Sidsel Karlsen**, and **Heidi Westerlund** illustrate the ways in which the multiple and intersecting identities of young people are often obscured through simplistic and homogenizing notions of popular music as “youth music” in Finland. Conducting an intersectional analysis of previous and current school curricula, they argue for a broader conceptualization of diversity at the policy level and the development of professional reflexivity and a “praxis of reflexivity” (Bubar et al. 2016) in music teacher education, in responding to the needs of an increasingly diverse and changing society. How discourses of diversity are mobilized in music education is the focus of the next chapter by **Elizabeth Gould**, who argues that much of this work supports and maintains the biopolitics of neoliberalism that upholds the privilege of white heteropatriarchy and feeds antidemocratic ends. Challenging the notion that *sameness* is a prerequisite of *equality*, she suggests that queer of color critique may equip music education researchers with perspectives to invest in people and musics that have thus far been overlooked or excluded in music education research and practice. Similarly extending the scope of diversity discourses in music education, **Vincent Bates**, **Anita Prest**, and **Daniel Shevock** situate music and education within a broader ecodiversity in approaching concerns of justice in a more holistic way. Considering how music education might attend to climate change, the destruction of ecosystems, and threats of extinction, the authors draw upon new materialism, political ecology, and indigenous knowledges, calling for a view that extends beyond the anthropocene while also nurturing local practices. Such an ecocentric approach to music education, they suggest, allows for a more sustainable, ethical way of musicking and living in the world.

The final section of the book turns to matters of leadership in higher music education, as an inherently political undertaking. There is an unprecedented demand upon institutions of higher education to respond to the current global climate of social demographic change, economic instability, technological advances, and crisis of social inequality. Such a response “inevitably forces examination of core values and brings to the fore the issues of ethics in higher education leadership” (Torrisi-Steele 2020, p. 2), in considering what higher education ought to be, why, what, or who, for. The discipline of music education is by no means exempt, and questions pertaining to the politics of diversity in music education leadership are arguably more acute and urgent than ever. For instance, as the recent crises faced by many higher education institutions, staff, and students in the wake of the novel coronavirus SARS-CoV-2 pandemic illustrate, dramatic changes have already taken place with regard to how international students are positioned in the university and to considerations of how institutions can meet the evolving needs of a diverse student body. In the opening chapter of this section, **Biranda Ford** interrogates the implicit colonialism of international recruitment policies and pedagogical practices of many Western conservatoires, raising questions as to how international students can be included as part of the educational community in more ethical and equitable ways. She challenges the positioning of international students as “in need” of a Western education and negative cultural stereotypes that shape the reception of their performance practices. Ford draws on the theories of Homi Bhabha (2006) in proposing that higher education can facilitate the decolonization of knowledge and culture through the creation of a “third space” in which transcultural dialogue can take place, forging a more sensitive and ethical relationship between institutions and international students. Considering what a sustainable global music education community might look like within the culturally sensitive internationalization of higher education institutions, **Alexandra Kertz-Welzel** offers critical considerations for leaders to promote intercultural understanding and a global mindset. Embracing the complexity and multiplicity of similarities and differences between musics, traditions, and cultures, she suggests that conceiving of the global music education community as symbolic and cosmopolitan may allow for a sense of unity while also respecting and cherishing diversity. In challenging the hegemonic dominance of any one music, language, or research culture, Kertz-Welzel invites us to reflect upon, and refine, our ideas of the community to which we belong and what we want this community to be in the future. In the final chapter of this section and the book, **Sidsel Karlsen** considers what might be required in the cultivation of such global higher music education communities, through an intercultural collaboration between higher music education institutions in Finland, Israel, and Nepal. She finds that institution leaders are required to perform complex negotiations between local and global discourses, navigating different values, traditions, hierarchies, as well as sociocultural and economic conditions. Karlsen critically examines the potential difficulties that may occur within and through collaborative processes, concluding that, paradoxically, “intercultural collaboration in higher music education might produce inequalities just as much as it aims for equality.” Together, the chapters of this final section of the volume posit a strong argument for more relational, culturally responsive, and context-specific approaches to higher music education leadership as the field works towards conceptualizing and engaging with diversity in more complex and ethical ways.

In sum, this book contributes towards a growing body of scholarship that reaches beyond a “happy image of diversity” (Ahmed 2012, p. 152) to a more critical, complex, and nuanced understanding of the ways in which the politics of diversity shape our ideals of what music education is, what it is for, and the actions we take in pursuing these ends in various contexts. If music education research, policy, and practice are to meet the needs of contemporary societies, it is essential for scholars and educators to continuously and critically examine the relationality, contextuality, and the ethics of such practice. Read together, the chapters of this book remind us that the ethical demands of music education resist approaches to the politics of diversity that are hinged upon finding “solutions” to diversity or the challenges that arise in diverse settings through rigid or all-encompassing rules, methods, or frameworks. *The Politics of Diversity in Music Education* thus serves as an invitation to ongoing reflexive inquiry; to deliberate the politics of diversity in a fast-changing and pluralist world; and together work towards more informed and ethically sound understandings of how diversity in music education policy, practice, and research is framed and conditioned both locally and globally.

## References

[CR1] Ahmed S (2012). On being included: Racism and diversity in institutional life.

[CR2] Ball A, Tyson CA, Ball AF, Tyson CA (2011). Preparing teachers for diversity in the twenty-first century. Studying diversity in teacher education.

[CR3] Banks JA (1993). Multicultural education: Development, dimensions, and challenges. The Phi Delta Kappan.

[CR4] Bauman Z (2010). 44 letters from the liquid modern world.

[CR5] Bhabha H (2006). The location of culture.

[CR6] Bubar R, Cespedes K, Bundy-Fazioli K (2016). Intersectionality and social work: Omissions of race, class, and sexuality in graduate school education. Journal of Social Work Education.

[CR7] Campbell PS, Sarath EW, Myers DE, Campbell PS (2017). The lay of the land. Redefining music studies in an age of change: Creativity, diversity, and integration.

[CR8] Campbell PS (2018). Music, education, and diversity: Bridging cultures and communities.

[CR9] Cousin G, Savin-Baden M, Howell C (2010). Positioning positionality: The reflexive turn. New approaches to qualitative research: Wisdom and uncertainty.

[CR10] Darling-Hammond L, Lieberman A, Darling-Hammond L, Lieberman A (2012). Teacher education around the world: What can we learn from international practice?. Teacher education around the world. Changing policies and practices.

[CR11] Dobusch L (2017). Diversity discourses and the articulation of discrimination: The case of public organizations. Journal of Ethnic and Migration Studies.

[CR12] Elliott DJ (1995). Music matters. A new philosophy of music education.

[CR13] Hakkarainen K, Gaunt H, Westerlund H (2013). Mapping the research ground: Expertise, collective creativity and shared knowledge practices. Collaborative learning in higher music education.

[CR14] Hesmondhalgh D (2013). Why music matters.

[CR15] Hess J (2015). Upping the “anti-”: The value of an anti-racist theoretical framework in music education. Action, Criticism, and Theory for Music Education.

[CR16] Jackson M (1989). Paths towards a clearing: Radical empiricism and ethnographic inquiry.

[CR17] Kallio AA, Westerlund H, Westerlund H, Karlsen S, Partti H (2020). The discomfort of intercultural learning in music teacher education. Visions for intercultural music teacher education.

[CR18] Kallio AA, Westerlund H, Partti H (2014). The quest for authenticity in the music classroom: Sinking or swimming?. Nordic Research in Music Education Yearbook.

[CR19] Karlsen S, Schmidt P, Colwell R (2017). Policy, access, and multicultural (music) education. Policy and the political life of music education.

[CR20] Karlsen S, Westerlund H, Benedict C, Schmidt P, Spruce G, Woodford P (2015). Music teachers’ repertoire choices and the quest for solidarity: Opening public arenas for the art of living with difference. The Oxford handbook of social justice in music education.

[CR21] Määttänen P, Westerlund H (2001). Travel agency of musical meanings? Discussion on music and context in Keith Swanwick’s interculturalism. British Journal of Music Education.

[CR22] Marcus GE, Fischer MM (1986). Anthropology as cultural critique.

[CR23] Marsh K, Ingram C, Dieckmann S, Westerlund H, Karlsen S, Partti H (2020). Bridging musical worlds: Musical collaboration between student musician-educators and south Sudanese Australian youth. Visions for intercultural music teacher education.

[CR24] Palmer AJ (1992). World musics in music education: The matter of authenticity. International Journal of Music Education.

[CR25] Pettan S, Clausen B, Hemetek U, Sæther E (2009). Europe and the potentials of music in motion. Music in motion: Diversity and dialogue in Europe.

[CR26] Sæther E, Houmann A, Sæther E (2020). Voice and agency in music education – Asking for societal networks. Make music matter: Music education meeting the needs of young learners.

[CR27] Schippers H (2010). Facing the music: Shaping music education from a global perspective.

[CR28] Stoller P (1987). In sorcery’s shadow. A memoir of apprenticeship among the Songhay of Niger.

[CR29] Stoller P (1989). The taste of ethnographic things: The senses in anthropology.

[CR30] Swanwick K (1988). Music, mind, and education.

[CR31] Swanwick K (1994). Musical knowledge: Intuition, analysis, and music education.

[CR32] Torrisi-Steele G, Wang V (2020). Ethics in higher education leadership: Current themes and trends. Handbook of research on ethical challenges in higher education leadership and administration.

[CR33] Vannini P, Williams JP (2009). Authenticity in culture, self, and society.

[CR34] Vertovec S, Vertovec S (2010). Super-diversity and its implications. Anthropology of migration and multiculturalism: New directions.

[CR35] Volk T (1998). Music, education, and multiculturalism.

[CR36] Westerlund H (2019). The return of moral questions: Expanding social epistemology in music education. Music Education Research.

[CR37] Westerlund H, Karlsen S (2017). Knowledge production beyond local and national blindspots: Remedying professional ocularcentrism of diversity in music teacher education. Action, Criticism, and Theory for Music Education.

[CR39] Westerlund H, Partti H (2018). A cosmopolitan culture-bearer as activist: Striving for gender inclusion in Nepali music education. International Journal of Music Education.

[CR40] Westerlund H, Partti H, Karlsen S (2015). Teaching as improvisational experience: Student music teachers’ reflections on learning during an intercultural project. Research Studies in Music Education.

[CR41] Westerlund H, Partti H, Karlsen S, MacDonald R, Miell D, Hargreaves D (2017). Identity formation and agency in the diverse music classroom. The Oxford handbook on musical identities.

[CR42] Westerlund H, Karlsen S, Partti H, Westerlund H, Karlsen S, Partti H (2020). Introduction. Visions for intercultural music teacher education.

